# Gene Families of Cuticular Proteins Analogous to Peritrophins (CPAPs) in *Tribolium castaneum* Have Diverse Functions

**DOI:** 10.1371/journal.pone.0049844

**Published:** 2012-11-21

**Authors:** Sinu Jasrapuria, Charles A. Specht, Karl J. Kramer, Richard W. Beeman, Subbaratnam Muthukrishnan

**Affiliations:** 1 Department of Biochemistry, Kansas State University, Manhattan, Kansas, United States of America; 2 Department of Medicine, University of Massachusetts, Worcester, Massachusetts, United States of America; 3 USDA-ARS Center for Grain and Animal Health Research Center, Manhattan, Kansas, United States of America; University of Kentucky, United States of America

## Abstract

The functional characterization of an entire class of 17 genes from the red flour beetle, *Tribolium castaneum*, which encode two families of Cuticular Proteins Analogous to Peritrophins (CPAPs) has been carried out. *CPAP* genes in *T. castaneum* are expressed exclusively in cuticle-forming tissues and have been classified into two families, *CPAP1* and *CPAP3*, based on whether the proteins contain either one (CPAP1), or three copies (CPAP3) of the chitin-binding domain, ChtBD2, with its six characteristically spaced cysteine residues. Individual members of the *TcCPAP1* and *TcCPAP3* gene families have distinct developmental patterns of expression. Many of these proteins serve essential and non-redundant functions in maintaining the structural integrity of the cuticle in different parts of the insect anatomy. Three genes of the *TcCPAP1* family and five genes of the *TcCPAP3* family are essential for insect development, molting, cuticle integrity, proper locomotion or fecundity. RNA interference (RNAi) targeting *TcCPAP1-C, TcCPAP1-H, TcCPAP1-J* or *TcCPAP3-C* transcripts resulted in death at the pharate adult stage of development. RNAi for *Tc*C*PAP3-A1, TcCPAP3-B, TcCPAP3-D1* or *TcCPAP3-D2* genes resulted in different developmental defects, including adult/embryonic mortality, abnormal elytra or hindwings, or an abnormal ‘stiff-jointed’ gait. These results provide experimental support for specialization in the functions of CPAP proteins in *T. castaneum* and a biological rationale for the conservation of CPAP orthologs in other orders of insects. This is the first comprehensive functional analysis of an entire class of cuticular proteins with one or more ChtBD2 domains in any insect species.

## Introduction

Chitin, a linear polymer of ß-1-4 linked N-acetyl-D-glucosamine, is an important component of the insect exoskeleton. It is present in epidermal and tracheal cuticles and in the peritrophic matrix (PM) that lines the midgut. Insect procuticle is composed mainly of chitin complexed with proteins that contain chitin-binding domains. Two major groups of chitin-binding domains found in insect cuticular proteins have either the Rebers & Riddiford Consensus sequence (R&R Consensus; pfam00379; [1]) that lacks cysteine residues, or the peritrophin-A motif with six distinctly spaced cysteine residues (ChtBD2 domain, pfam 01607). Proteins with the ChtBD2 domain were found initially in extracts from insect PMs [2]. Additional genes encoding proteins with the ChtBD2 domain were found to be expressed in cuticle-forming tissues from *Drosophila melanogaster* and *Ctenocephalides felis* [3], [4], [5], suggesting that the peritrophin-A motif is not restricted to proteins associated only with the PM.

By conducting a bioinformatics search of the *Tribolium castaneum* (red flour beetle) genome, we identified 49 putative genes capable of encoding 50 proteins with one or more ChtBD2s [6]. These include several enzymes of chitin metabolism as well as several other proteins with 1–14 ChtBD2 domains. Almost all of them are predicted to have a cleavable signal peptide, suggesting that they are secreted and potentially capable of interacting with extracellular chitin. Among these 50 proteins, 11 were expressed in gut tissues but not in the carcass, and therefore are likely to be associated with chitin in the PM. These have been classified as **P**eritrophic **M**atrix **P**roteins (PMPs) [6]. Included among the remaining 39 predicted proteins with ChtBD2 motifs were two distinct families of proteins that were expressed only in cuticle-forming tissues but not in the midgut. We have named these proteins “**C**uticular **P**rotein **A**nalogous to **P**eritrophins” (CPAPs).

Members of these two families of proteins have either one or three ChtBD2 domains but no other identifiable protein domains. We have classified them as belonging to the CPAP1 family (ten proteins encoded by ten genes) each with only one ChtBD2 domain, or the CPAP3 family (eight proteins encoded by seven genes) each with three ChtBD2 domains. The CPAP1 family of proteins are of variable length and show amino acid sequence conservation only in the ChtBD2 domain that typically constitutes only a small fraction of the total length of each protein. In contrast, each of the CPAP3 family proteins consists of three N-terminal tandem copies of the ChtBD2 domain, separated by small conserved linker regions and a short, but variable, C-terminal region.

Members of the CPAP3 family of proteins are encoded by orthologs of *D. melanogaster* genes collectively known as the “*gasp*” or “*obstructor*” family [3], [5]. We have assigned names to individual *T. castaneum* members of this family that reflect their orthology to the corresponding *D. melanogaster obstructor* gene (e.g. *T. castaneum CPAP3-A* is the ortholog of *D. melanogaster Obst-A*). Homologs belonging to a separate subgroup (subgroup II) consisting of *obstructor*s F, G, H, I and J were also identified in *D. melanogaster* [3], but these are absent from the genome of *T. castaneum* [6]. Thus, there are five fewer members of the *CPAP3* family in *T. castaneum* than in *D. melanogaster*.

Phylogenetic analysis of the *T. castaneum* CPAP proteins revealed that CPAP1 and CPAP3 represent two distinct branches of a tree that also includes a large assortment of peritrophins that have related ChtBD2 domains [6]. Genes encoding orthologs of the eight members of the CPAP3 family of proteins (CPAP3-A1, -A2, -B, -C5a, -C5b, -D1, -D2 and -E) are found in insects belonging to several different orders whose genomes have been fully sequenced and annotated, suggesting that these proteins are essential for insect survival or fitness. On the other hand, orthologs of the *T. castaneum* genes encoding CPAP1 proteins have been found in only some of those insect orders, perhaps suggesting that they are less critical for insect survival (Table 1).

**Table 1:** Identification of orthologs of CPAP1 and CPAP3 proteins in phyla Arthropoda and Nematoda.

		PHYLUM: ARTHROPODA								
		SUB-PHYLUM: HEXAPODA								
		CLASS: INSECTA								
CPAPs	Accession #	Diptera	Coleoptera	Hymenoptera	Hemiptera	Phthiraptera	Lepidoptera	Crustacea	Chelicerata	Nematoda*
**CPAP3-A1**	ACY95475	**+**	**+**	**+**	**+**	**+**	id	**+**	**−**	**−**
**CPAP3-A2**	ACY95476	**+**	**+**	**+**	**+**	**+**	id	**+**	**−**	**−**
**CPAP3-B**	ABL73928	**+**	**+**	**+**	**+**	**id**	**+**	**+**	**−**	**−**
**CPAP3-C**	ABL73929	**+**	**+**	**+**	**+**	**+**	**+**	**+**	**−**	**−**
**CPAP3-D1**	ACY95477	**+**	**+**	**+**	**+**	**+**	**+**	**+**	**+**	**−**
**CPAP3-D2**	ABL73931	**+**	**+**	**+**	**+**	**+**	**id**	**+**	**+**	**−**
**CPAP3-E**	ACY95478	**+**	**+**	**+**	**+**	**+**	**id**	**+**	**−**	**−**
**CPAP1-A**	ACY95466	**−**	**+**	**+**	**+**	**−**	**−**	**−**	**−**	**−**
**CPAP1-B**	ACY95467	**+**	**+**	**+**	**−**	**−**	**id**	**−**	**−**	**−**
**CPAP1-C**	ACY95468	**+**	**+**	**−**	**−**	**−**	**−**	**−**	**−**	**−**
**CPAP1-D**	ACY95469	**+**	**+**	**+**	**−**	**id**	**−**	**−**	**−**	**−**
**CPAP1-E**	ACY95470	**−**	**+**	**−**	**−**	**id**	**−**	**+**	**−**	**−**
**CPAP1-F**	ACY95471	**−**	**+**	**+**	**−**	**−**	**−**	**−**	**−**	**−**
**CPAP1-G**	ACY95472	**+**	**+**	**+**	**−**	**id**	**id**	**−**	**−**	**−**
**CPAP1-H**	ACY95473	**+**	**+**	**−**	**−**	**id**	**−**	**−**	**−**	**−**
**CPAP1-I**	ACZ04319	**−**	**+**	**−**	**−**	**id**	**id**	**−**	**−**	**−**
**CPAP1-J**	ACY95474	**−**	**+**	**−**	**−**	**id**	**id**	**−**	**−**	**−**

Representative members for different insect orders, sub-phyla and phyla: *Diptera- Drosophila, Culex, Anopheles, Aedes, Glossina, Saptomyza, Lutzomiya, Culicoides; Lepidoptera- Choristeneura, Bombyx, Spodoptera, Loxostege, Tenebrio, Mamestra, Helicoverpa, Heliconius, Plutella; Coleoptera- Tribolium, Holotrichia, Popillia, Tenebrio; Hymenoptera- Harpegnathus, Nasonia, Solenopsis, Apis, Acromyrmex, Bombus, Camponotus; Hemiptera- Acyrthosiphon; Phthiraptera- Pediculus; Crustacea- Daphnia, Artemia, Lepeophtheirus, Caligus, Rimicaris; Chelicerata- Ixodes;* Nematoda- *Ascaris*, *Caenorhabditis.* id: insufficient data; “+”if overall similarity had e-values greater than >e^−22^; “−”if overall similarity had e-values less than <e^−22^. “*”In Nematoda indicates the presence of putative homologs of CPAP proteins in *C. elegans* and *Brugia malayi.* The *Brugia malayi* protein XP_001900473 has three ChtBD2’s but it is more closely related to the peritrophic matrix protein consensus of the ChtBD2 families [6]. Another *C. elegans* protein CEJ-1 with 3 ChtBD2 has interspersed mucin domains. However, their orthology to the CPAP3 family proteins is undetermined.

All members of the *CPAP1* and *CPAP3* families of *T. castaneum* genes are expressed in cuticle-forming tissues, but not in the PM-secreting midgut [6]. Furthermore, there are differences in the relative expression levels of these genes in two closely related cuticle-forming tissues with different mechanical properties, namely the elytron and the hindwing, indicating tissue-specific regulation of expression of *CPAP* genes [6]. Corresponding differences in tissue-specific expression of the *obstructor* family of genes in *D. melanogaster* (orthologs of *TcCPAP3* family) have been reported previously [3]. Recently, a detailed study on the functions of one member of this family, *obstructor A*, from *D. melanogaster* has been carried out [7]. That study has shown that this gene is essential for embryonic development and affects tracheal tubule formation by preventing premature turnover of lumenal chitin. In this study, we have carried out an extensive expression and functional analyses of *T. castnaeum* genes encoding each of the eighteen proteins belonging to the *CPAP1* or *CPAP3* families in order to explore their roles in cuticle formation and integrity, insect survival and fecundity at different developmental stages. The results of RNAi experiments indicate that many of these genes have distinct and non-redundant essential functions in development and in maintenance of the structural integrity of cuticles with a wide range of physical properties.

## Materials and Methods

### Insect Cultures

The *T. castaneum* strains used were the standard wild-type GA-1 and *pu11*, an enhancer trap line in which the gene for ­enhanced green fluorescent protein (EGFP) is expressed in the wing and elytral discs at the pharate pupal stage, but not in earlier stages [8]. In *T. castaneum,* the different larval instars cannot be reliably distinguished. To circumvent this problem, the *pu11* strain was used to enable the identification of animals in the penultimate or earlier larval instar and facilitate RNAi experiments at desired developmental stages. Insects were reared at 30°C in wheat flour containing 5% brewer’s yeast under standard conditions as described previously [9].

### Developmental Expression Profiles of the CPAP Gene Families

The developmental expression patterns of the ten *CPAP1* genes and seven *CPAP3* genes were analyzed by RT-PCR. Total RNA was isolated by using the RNeasy Mini kit (Qiagen, Valencia, CA) according to the manufacturer’s instructions. RNA sources included whole insects at various stages of development including embryos, penultimate instar larvae, last instar larvae, pharate pupae, pupae, young adults (0–3 hours after adult eclosion) and mature adults (10 days-old), as well as pupae collected at day-0 to day-5 post-pupation. First strand cDNA synthesis was carried out using the Superscript III first-strand synthesis system (Invitrogen, Carlsbad, CA) utilizing 1 µg of total RNA for each reaction. This cDNA served as template for subsequent PCR reactions using the gene-specific primers listed in Jasrapuria et al., [6].

### Double-stranded RNA Synthesis and Injection

Double-stranded RNAs (dsRNAs) were synthesized from the PCR-generated cDNA templates using the Ampliscribe™ T7-Flash™ Kit (Epicentre Technologies, Madison, WI) as per the manufacturer’s protocol. Unique regions with greatest sequence divergence for each of the *TcCPAP1* and *TcCPAP3* genes were chosen as templates for synthesis of dsRNAs. Their positions in the target gene and sizes are indicated in Supplementary Table S1. In most cases, RNAi experiments were replicated by choosing two non-overlapping regions as targets. To assess the functional specificity of the two isoforms of proteins encoded by *TcCPAP3-C*, dsRNAs corresponding to the entire sequence of either alternative exon 5a or 5b of this gene were synthesized. Double-stranded RNAs (200 ng per insect in 0.1 mM potassium phosphate buffer containing 5 mM KCl, pH 7) corresponding to each target gene region were injected into either penultimate or last instar larvae (n = 40 for each stage) as described previously [10]. A dsRNA for the *T. castaneum* tryptophan oxygenase gene (*TcVermilion, TcVer*) was used as a positive control for effectiveness of RNAi as well as a negative control for the effects of a non-target dsRNA. DsRNA for *chitin synthase A* (*Chs-A*), which produces a lethal phenotype at the pharate adult stage, was used as a non-target gene control to determine if transcripts for this gene were affected by the dsRNA treatments.

To observe any effects of RNAi on fecundity, oviposition behavior or embryonic development, dsRNAs for each of the ten *TcCPAP1* genes and the seven *TcCPAP3* genes were injected singly into adult females (n = 20). Two days after injection, dsRNA-treated females were mated with an equal number of untreated adult males. These insects were maintained under normal conditions as described [11]. Eggs were collected every 3 days for ∼1 month for determining fecundity, hatchability and chitin content. Eggs were dechorionated by treating with 1∶1 diluted 5% Clorox solution before recording images using a Leica MZFLIII microscope.

### Measurement of Transcripts after RNAi for CPAP Families of Genes

RT-PCR experiments were carried out to monitor target-specific transcript depletion. Total RNA was isolated from whole insects 4 days after dsRNA injection using the RNeasy Mini kit (Qiagen, Valencia, CA). Three insects were pooled for each RNA extraction. cDNA synthesis and RT-PCR were performed using gene-specific primer pairs. The specificity of RNAi for the targeted *CPAP* genes was confirmed by comparing the transcript levels of both the targeted gene, several closely related non-targeted *TcCPAP1* or *TcCPAP3* genes, as well as the *Chs-A* gene. PCR amplification products using the same cDNA template and a pair of primers for the gene encoding ribosomal protein S6 (RpS6) were used as internal loading controls.

### Scanning Electron Microscopy (SEM) of Elytra after RNAi for TcCPAP3-D

Co-injection of dsRNAs for the *TcCPAP3-D1* and *TcCPAP3-D2* genes (200 ng of each dsRNA per insect; n = 6) was done at the pharate pupal stage to maximize the effect of RNAi of these paralogous genes. Elytra were dissected 10-days after adult eclosion and fixed by passing through a series of washing solutions and subjected to critical point drying. The elytral samples were coated with a 60% gold/40% palladium mixture using a DESK II Sputter/Etch unit (Denton Vacuum, LLC, Moorestown, NJ). Images were taken using the S-3500N scanning electron microscope equipped with the S-6542 Absorbed Electron Detector (Hitachi Science Systems, Ltd., Hitachinaka, Japan).

### Chitin Staining of Elytra with FITC-CBD after RNAi for CPAP Genes

dsRNA injections into pharate pupae were repeated for each *CPAP* gene that was shown by RNAi experiments to be required for normal adult eclosion. These included *TcCPAP1-C*, *TcCPAP1-H*, *TcCPAP1-J* and *TcCPAP3-C* with *TcChs-A* and *TcVer* serving as positive and negative controls, respectively. Elytra were dissected from pharate adults, washed in 1×PBS, pH 8, and then heated in 10 M NaOH for 6 hours at 95°C to remove the proteins. The resulting mixture with the insoluble cuticular material was neutralized in 1×PBS, pH 8, and stained at room temperature for 1 hour with a chitin probe consisting of the fluorescein isothiocyanate-conjugated chitin-binding domain from chitinase of *B. circulans* W12 (FITC-CBD, New England BioLabs, Beverly, MA) at a 1∶500 dilution in 1×PBS, pH 8. After washing off the excess FITC-CBD probe with 1×PBS, the green fluorescence was recorded using a GFP filter (470 nm excitation and 515 nm emission wavelengths) and a Leica MZFLIII microscope.

### Chitin Content Analysis of Insects after RNAi for CPAP1 and CPAP3 Family Genes

Double-stranded RNA for each of the *TcCPAP1* or *TcCPAP3* genes was injected into insects at the pharate pupal stage. The treated insects were collected 5 days post-pupation at the pharate adult stage just prior to adult eclosion. Individual pharate adults were weighed and placed in separate 1.5 ml screw-cap microcentrifuge tubes. The total chitin content of each of these insects was measured using a modified Morgan-Elson method as described previously [12].

### Confocal Microscopic Analysis of dsRNA CPAP1-H-treated Insects

*TcCPAP1-H* dsRNA-injected pharate pupae were collected at pupal day 5. The insects were fixed in 4% p-formaldehyde and passed through a sucrose gradient series. Cryosectioning was performed as described previously [13] using a Leica CM 1800 instrument. Twenty µm thick cross-sections of pharate adults (pupal day-5) were stained for chitin with the rhodamine-conjugated chitin-binding probe (New England Biolabs, Beverly, MA). All incubations for immunofluorescence were performed at room temperature. The sections were rinsed three times for 5 min in 0.2% PBST buffer, incubated for 1 hour in PBST buffer containing 2% BSA, and then rinsed. The sections were incubated overnight with rhodamine-conjugated chitin binding probe (1∶100 dilution) in 2% BSA. After this treatment, the sections were rinsed again with PBST buffer as described above and nuclei were stained with 4', 6-diamidino-2-phenylindole-dihydrochloride (DAPI, 1 mg/ml) in PBS buffer. After further washing with PBST buffer, the sections were covered with Fluromount (Sigma) and sealed with clear nail polish to prevent dehydration. Slides were visualized using a Zeiss Leica 510 confocal microscope. The instrument with a fully motorized stage, a Plan Apochromat objective (40 X/1.4 oil) and differential contrast interference (DIC) was used for taking images. Fluorescence emission imaging of the 543 nm line of the He-Ne laser was used to excite the rhodamine-conjugated chitin-binding probe and the 405 nm line for DAPI staining. Analysis of the images was done using the NIH Image J software.

## Results

**Figure 1:**
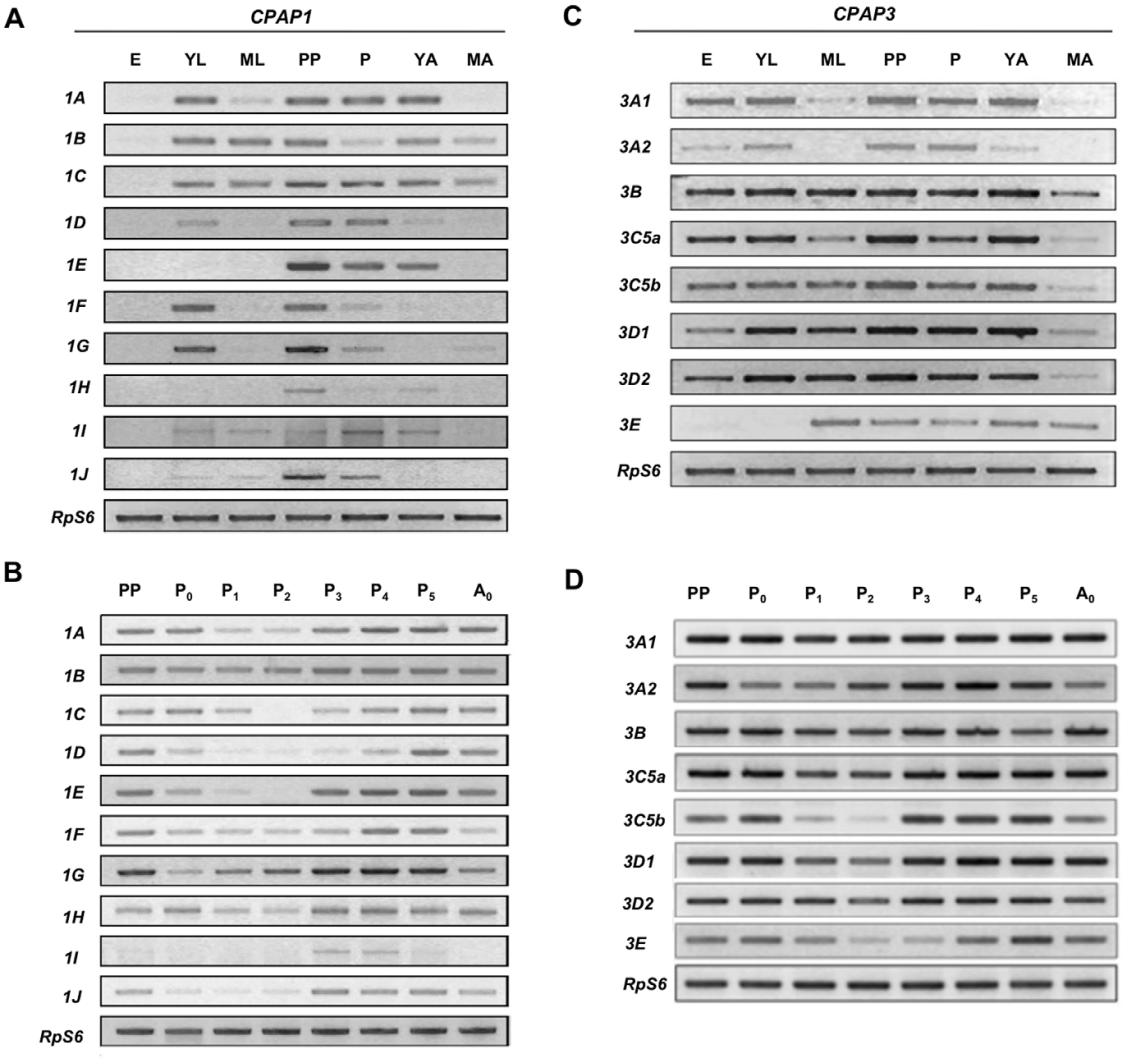
Expression profiles of members of *CPAP* gene families (*CPAP1* and *CPAP3*) as determined by RT-PCR. Approximately 500 eggs (14.9 mg) were collected and used for RNA isolation. Pools of four larvae, (young and old separately) pharate pupae, pupae from day-0 to day-5, young adults (∼1–2 hour old) or mature adults (>2 weeks-old) were used for preparation of total RNA at these stages. cDNAs synthesized from total RNA using the oligo-(dT) primer and reverse transcriptase were used as templates for RT-PCR (28 cycles).The initial denaturation was at 94°C for 10 minutes, annealing at (56°C–60°C) for 1 minute and the final extension was at 72°C for 10 minutes, using gene-specific primers. RT-PCR products for the *T. castaneum* ribosomal protein S6 gene (*RpS6*) from the same cDNA templates served as an internal control for loading (24 cycles). (A) Expression profiles of *CPAP1* genes at different stages of development: E, eggs; YL, penultimate instar or younger larvae; ML, mature (last instar) larvae; PP, pharate pupae; P, pupae; YA, young adult; 3 hours after eclosion; MA, mature adult; 10 days after eclosion. (B) Expression profiles of *CPAP1* genes at different stages of pupal development. P_[0–5]_, pupae 0-day old to pupae 5-days old were collected at 1-day intervals. (C) Expression profiles of *CPAP3* genes at different stages of development: E, eggs; YL, penultimate instar or younger larvae; ML, mature (last instar) larvae; PP, pharate pupae; P, pupae; YA, young adult; 3 hours after eclosion; MA, mature adult; 10 days after eclosion. (D) Expression profiles of *CPAP3* genes at different stages of pupal development. P_[0–5]_, pupae 0-day-old to pupae 5-days-old were collected at 1-day intervals.

As a first step in the functional analysis of individual members of the *CPAP* gene families and optimization of RNA interference experiments, the expression profiles of the genes belonging to the two *CPAP* families of *T. castaneum* were determined by RT-PCR using cDNA prepared from RNA isolated at different stages of development. The results are shown in Fig. 1 and described below.

### CPAP1 Genes Exhibit Divergence in Expression Profiles

The individual genes of the *CPAP1* family have divergent expression patterns (Fig. 1A). The most notable finding is that they are expressed at very low levels, if at all, during the embryonic stage. *TcCPAP1-A*, *TcCPAP1-I* and *TcCPAP1-J* are expressed throughout the larval and pupal stages, with little or no expression in the adult. *TcCPAP1-B* and *TcCPAP1-C* have the broadest range of expression, spanning from young larval to mature adult stages. Others have more restricted ranges of expression. For example, *TcCPAP1-D*, *TcCPAP1-F* and *TcCPAP1-G* are expressed only in young larval and pupal stages. *TcCPAP1-E* and *TcCPAP1-H* are expressed predominantly in the pupal stage and do not appear to be expressed in earlier developmental stages or at the mature adult stage.

As most of the *CPAP1* genes show expression in the pharate pupal and pupal stages, more detailed expression patterns of *CPAP1* genes were also analyzed by RT-PCR from the pharate pupal stage through the young adult stage. *TcCPAP1-A*, *TcCPAP1-B*, *TcCPAP1-F*, *TcCPAP1-G* and *TcCPAP1-H* transcripts were observed throughout the pupal stage. *TcCPAP1-C* was expressed similarly, except for pupal day-2 when it had low or undetectable levels of expression. *TcCPAP1-D* had low expression on pupal days 1–3. *TcCPAP1-E* and *TcCPAP1-J* showed late pupal expression, but the transcript levels were relatively low during pupal days 0–3. *TcCPAP1-I* had very low expression throughout, except for detectable transcripts on pupal day-3 and pupal day-4 (Fig. 1B).

### CPAP3 Genes have Similar Expression Profiles Throughout Development

In contrast to the widely diverging patterns of expression among *CPAP1* genes, many of the *CPAP3* genes were expressed throughout all stages of development, from embryonic to young adult stages, at nearly the same levels, followed by low or undetectable levels at the mature adult stage (Fig. 1C). Notable exceptions were *TcCPAP3-A1* and *TcCPAP3-A2* with low levels of expression in the mature larval stage, and also *TcCPAP3-E* whose expression occurred mainly from the mature larval stage and continued into the mature adult stage. Expression analysis of the *CPAP3* genes from the pharate pupal to young adult stages revealed that all of these genes were expressed throughout this period except for *TcCPAP3-C5b* and *TcCPAP3-E*, both of which exhibited low levels of transcripts on pupal days 1–2 and pupal days 2–3, respectively (Fig. 1D).

### Specificity of dsRNA-mediated Transcript Depletion within Each Family

**Figure 2:**
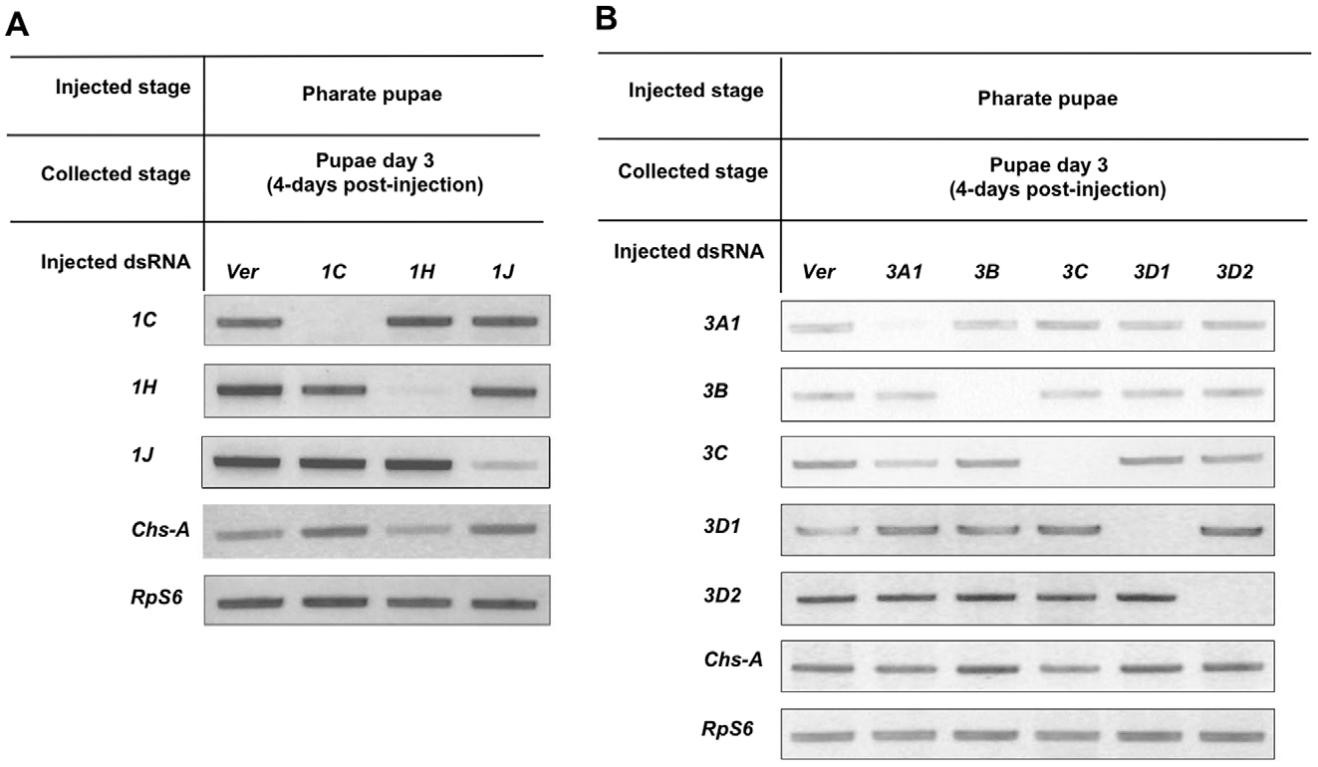
RT-PCR analyses of target-specificity of RNAi mediated by dsRNAs for *CPAP1* and *CPAP3* genes. RT-PCR was carried out using RNA isolated four days after dsRNA injections as template to monitor the extent of depletion of the transcripts of the targeted *CPAP* gene as well as some of the closely related gene(s) of the same family. The RT-PCR (28 cycles) results indicated significant reduction in the transcripts of the targeted *CPAP* gene with no evidence of depletion of transcripts of other genes of the same family. (A) Specificity of transcript knock-down after injection of dsRNA for *CPAP1* genes. (B) Specificity of transcript knock-down after treatment with dsRNA for *CPAP3* genes.

Sequence conservation among members of the CPAP1 family proteins is limited to the peritrophin-A domain, with the rest of the proteins being variable in sequence and in length. Therefore, there were no difficulties in locating regions in the cDNA sequences for the synthesis of dsRNAs unique for each *CPAP1* gene. RT-PCR following *CPAP1* RNAi indicated that there was no evidence of knockdown of non-targeted transcripts (see Fig. 2A for some examples). CPAP3 family proteins are more highly conserved, not only in the three peritrophin-A coding domains, but also in the two linker regions between these domains. However, we were still able to design dsRNAs unique to each member of the *CPAP3* gene family. The specificity of knock-down of the targeted *CPAP3* transcripts by dsRNAs for individual members of this family was confirmed by RT-PCR (Fig. 2B). Again down-regulation was confined to the targeted gene.

### Some CPAP1 Genes are Required for the Pupal-to-adult Molt

**Figure 3:**
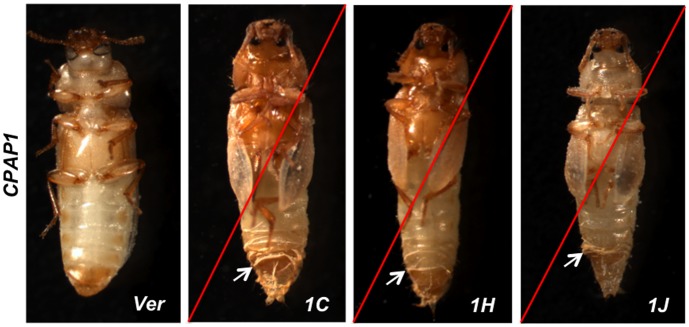
Treatment with dsRNA for *TcVer*, *TcCPAP1-C, TcCPAP1-H* or *TcCPAP1-J* affects pupal and adult development. The phenotypes produced by injection of dsRNAs for *CPAP1* genes at the penultimate and/or last instar larval stages are shown along with the phenotype of the *Vermilion (Ver)* dsRNA-injected control. Gene-specific dsRNAs for each of the three indicated *CPAP1* genes were injected into penultimate instar larvae (200 ng per insect; n = 40). The terminal phenotypes of insects after RNAi for *TcCPAP1-C, TcCPAP1-H* and *TcCPAP1-J* are shown. A red slash across the figure indicates that these are phenotypes of moribund animals. They all molted normally during earlier stages but exhibited developmental arrest at the pupal to adult molt and 100% lethality. White arrows indicate incomplete cuticle slippage. dsRNA *TcVer*-treated controls developed into adults with no mortality and had no abnormalities except for the expected loss of eye pigmentation.

To determine the roles of individual members of the two families of *CPAP* genes in insect development, we carried out RNAi experiments using dsRNAs specific for each gene. Our RNAi strategy was designed to down-regulate transcript levels for specific members of each *CPAP* gene family just prior to the stage(s) of peak expression of the target gene and/or prior to each molt. dsRNAs for each of the ten *CPAP1* genes were injected into insects (n = 40 for each group) at multiple stages of development, namely, penultimate instar larval, last instar larval, pharate pupal or pupal day 0 stages depending on the developmental expression profile of the target gene. The phenotypes of the insects subjected to *CPAP1* RNAi as well as insects injected with the dsRNA for *Vermilion* (ds*Ver*), which served as positive and negative controls, were monitored daily. Of the ten *CPAP1* family genes**,** *TcCPAP1-C*, *TcCPAP1-H* and *TcCPAP1-J* exhibited lethal phenotypes at the pharate adult stage, regardless of the stage at which dsRNA was injected (larval, pharate pupal and or pupal stages). At the time of developmental arrest, incomplete cuticle slippage was evident, and elytral expansion was incomplete, as shown in Fig. 3. The dsRNA *TcVer-*injected control insects developed into normal adults except for the lack of eye pigmentation [14]. Several members of the *CPAP3* family of genes were found to be essential for normal insect development and/or survival. In contrast to the results for *CPAP1* family genes, RNAi for many of the *CPAP3* family genes led to a range of molting defects, abnormal cuticles and lethality of insects at different stages of development. These abnormalities are described below for each member of the *CPAP3* family of genes of *T. castaneum*.

### RNAi Phenotypes of Members of the CPAP3 Gene Family

#### TcCPAP3-A1

**Figure 4:**
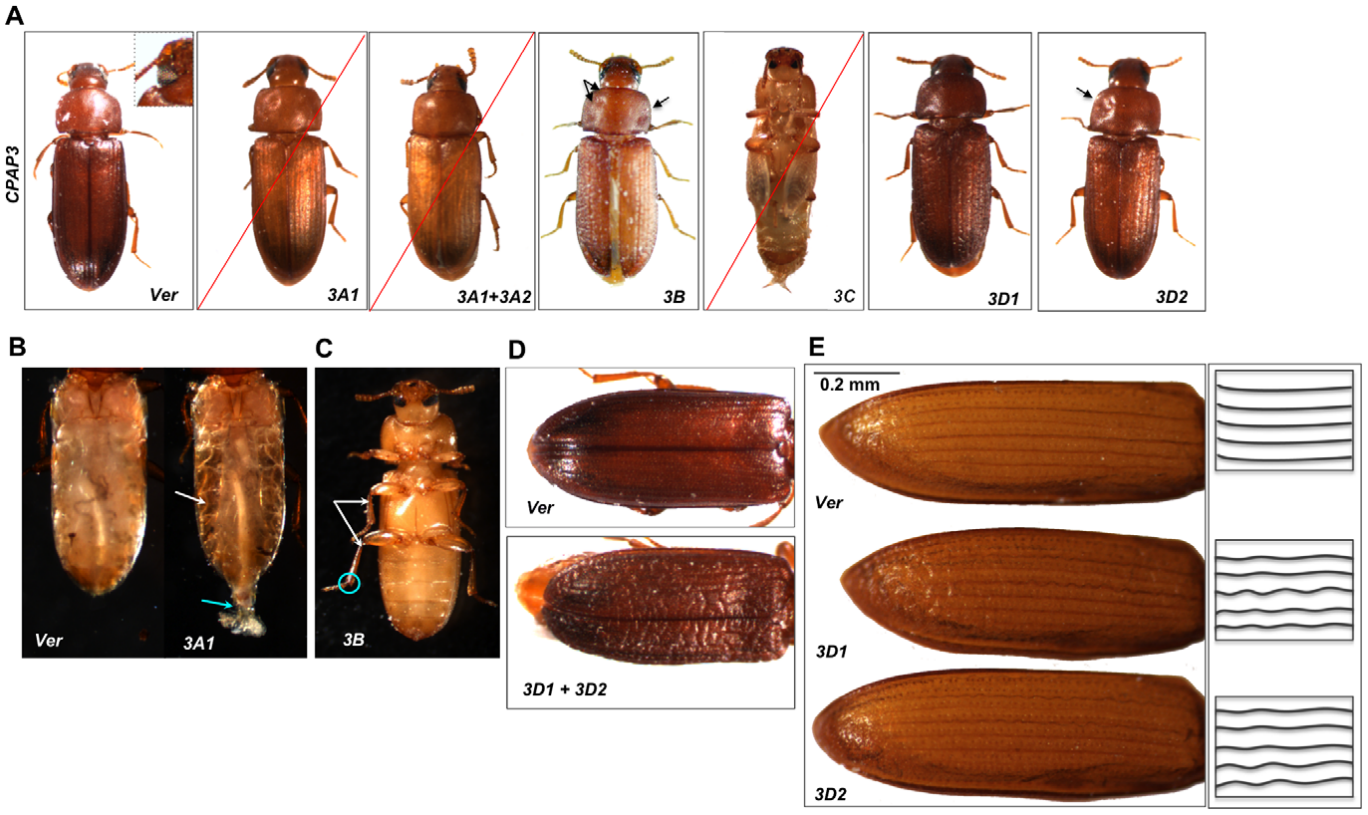
Summary of phenotypes after RNAi of *CPAP3* genes. (A) Insects injected with dsRNA at the penultimate and/or last instar larval stages for many of the *CPAP3* genes showed abnormal phenotypes (except for *TcCPAP3-A2* and *TcCPAP3-E).* RNAi for *TcCPAP3-A1* resulted in adult lethality (indicated by a red slash across the figure). dsRNA *TcCPAP3-A1+* dsRNA *TcCPAP3-A2:* the insects did not show additive mortality or additional abnormalities compared to animals injected with dsRNA *TcCPAP3-A1* alone; dsRNA *TcCPAP3-B:* adults show walking defects. Black arrows indicate dimpled pronotum. dsRNA *TcCPAP3C:* Insects showed pupal to adult molt arrest and 100% lethality. dsRNA *TcCPAP3-D1:* Insects had rough elytra, which did not completely cover the adult abdomen. dsRNA *TcCPAP3-D2*: The phenotype was similar to animals treated with dsRNA *TcCPAP3-D1,* but was less severe. dsRNA *TcVer*-injected insects (200 ng per insect; n = 40) were used as controls (white eye is shown in inset). (B) dsRNA *TcCPAP3-A1* treatment at the larval stages resulted in fat body depletion in one week-old adults compared to dsRNA *TcVer-*treated controls; shown by white arrows. Also the insects were unable to completely expel the fecal pellet; marked with blue arrow. (C) dsRNA *TcCPAP3-B* treated adults show a wobbly, stiff-jointed gait, resulting from abnormal articulation of the tibial-tarsal (shown in blue circle) and femoral-tibial joints for the metathoracic legs and of the femoral-tibial joint for the mesothoracic legs (shown as white arrows). (D) dsRNAs for *TcCPAP3-D1* and *TcCPAP3-D2* were co-injected into pharate pupae (n = 40). The resulting insects molted to otherwise normal adults with abnormal elytra. The elytra were wrinkled and alligator skin-like, and did not completely cover the abdomen, but none of the insects died. dsRNA *TcVer* was injected as a control and the elytra from these insects were normal (top panel). (E) Defects in a single elytron following RNAi for *TcCPAP3-D1* or *TcCPAP3-D2* in comparison to the *TcVer* dsRNA treatment. The differences are also shown in the form of the schematic line diagram on the right, where the lines are wavy in comparison to the control, *TcVer.*

dsRNA-injected insects (n = 40; two dsRNA’s targeting different regions of the gene) exhibited a lethal phenotype only at the mature adult stage approximately one week after adult eclosion, regardless of the stage at which dsRNA was injected (larval, pharate pupal or pupal). These adults were unable to dislodge fecal pellets from their anuses and had severely depleted fat bodies (Fig. 4A, 4B). No other visible abnormalities were detected.

#### TcCPAP3-A2

RNAi for *TcCPAP3-A2* did not result in any observable abnormality at any stage of development. Thus this gene appears to be dispensable, in contrast to its paralog, *TcCPAP3-A1*. Furthermore, co-injection of dsRNAs for *TcCPAP3-A1* and *TcCPAP3-A2* led to the same phenotypes as those observed after ds*TcCPAP3-A1* injection alone, ruling out any synergism between these two genes (Fig. 4A).

#### TcCPAP3-B

Injection of dsRNA (n = 40) at the larval stage had no effect on pupation, adult eclosion or on adult mortality for up to two months. However, all resulting adults were permanently afflicted with a stiff, uncoordinated gait. The forelegs appeared to be least affected, whereas the second pair of legs showed normal tibial-tarsal joint movement but stiffness at the femoral-tibial joint (Fig. 4C). The hindlegs were most affected, with impaired articulation at both the femoral-tibial and tibial-tarsal joints, resulting in a severe walking disability as indicated by a wobbly gait (see Video S1). Nearly half of the eclosed adults also exhibited split elytra, which were rough and bumpy. The pronota were also dimpled (Fig. 4A).

#### TcCPAP3-C

Insects injected with dsRNA for *TcCPAP3-C* at either the larval or prepupal stage suffered developmental arrest and death at the pupal-to-adult molt. None of the insects (n = 40) were able to shed their pupal cuticle. On the other hand, injections of dsRNA specific for *TcCPAP3-C5a* or *TcCPAP3-C5b* exons corresponding to the two alternatively spliced forms of the fifth exon of the *TcCPAP3-C* gene [6] resulted in developmental arrest and death of only 5% and 10% of the insects, respectively. The moribund insects had a phenotype similar to that observed after administration of exon-non-specific dsRNA for *TcCPAP3-C*. This result indicated that these two alternatively spliced transcripts at least partially compensated for each other (Fig. 4A). Co-administration of splice variant-specific dsRNAs for both alternative exon resulted in a phenotype identical to that seen with RNAi using a dsRNA for a common exon (data not shown).

#### TcCPAP3-D1 *and* TcCPAP3-D2

Injection of dsRNAs for either *TcCPAP3-D1* (n = 40) or *TcCPAP3-D2* (n = 40) had no effect on larval-larval, larval-pupal or pupal–adult molts. However, there were visible differences in the appearance of elytra compared to dsRNA *TcVer*-treated controls. Insects injected with dsRNA for *TcCPAP3-D1* or/and dsRNA for *TcCPAP3-D2* failed to fully expand their elytra and the elytral surface was uneven and wrinkled (Fig. 4A, 4D and 4E). Also the pronota of dsRNA *TcCPAP3-D2*-treated insects were dimpled. Co-injection of dsRNAs for the paralogous *TcCPAP3-D1* and *TcCPAP3-D2* genes resulted in adult elytra that were more severely affected than animals injected with dsRNA for either gene alone, indicating synergism between these two genes (Fig. 4D), but the adults were still viable. The elytra had alligator skin-like wrinkles and creases, and were shorter in length compared to the dsRNA *TcVer*-injected controls that developed normally. RT-PCR tests to check transcript down-regulation after RNAi showed significant depletion of the *TcCPAP3-D1* and *TcCPAP3-D2* transcripts at the pharate adult stage relative to controls that were injected with dsRNA for *TcVer* (data not shown).

### Treatment with dsRNA for TcCPAP3-D1 or TcCPAP3-D2 Affects Morphology of Elytral Cuticle

**Figure 5:**
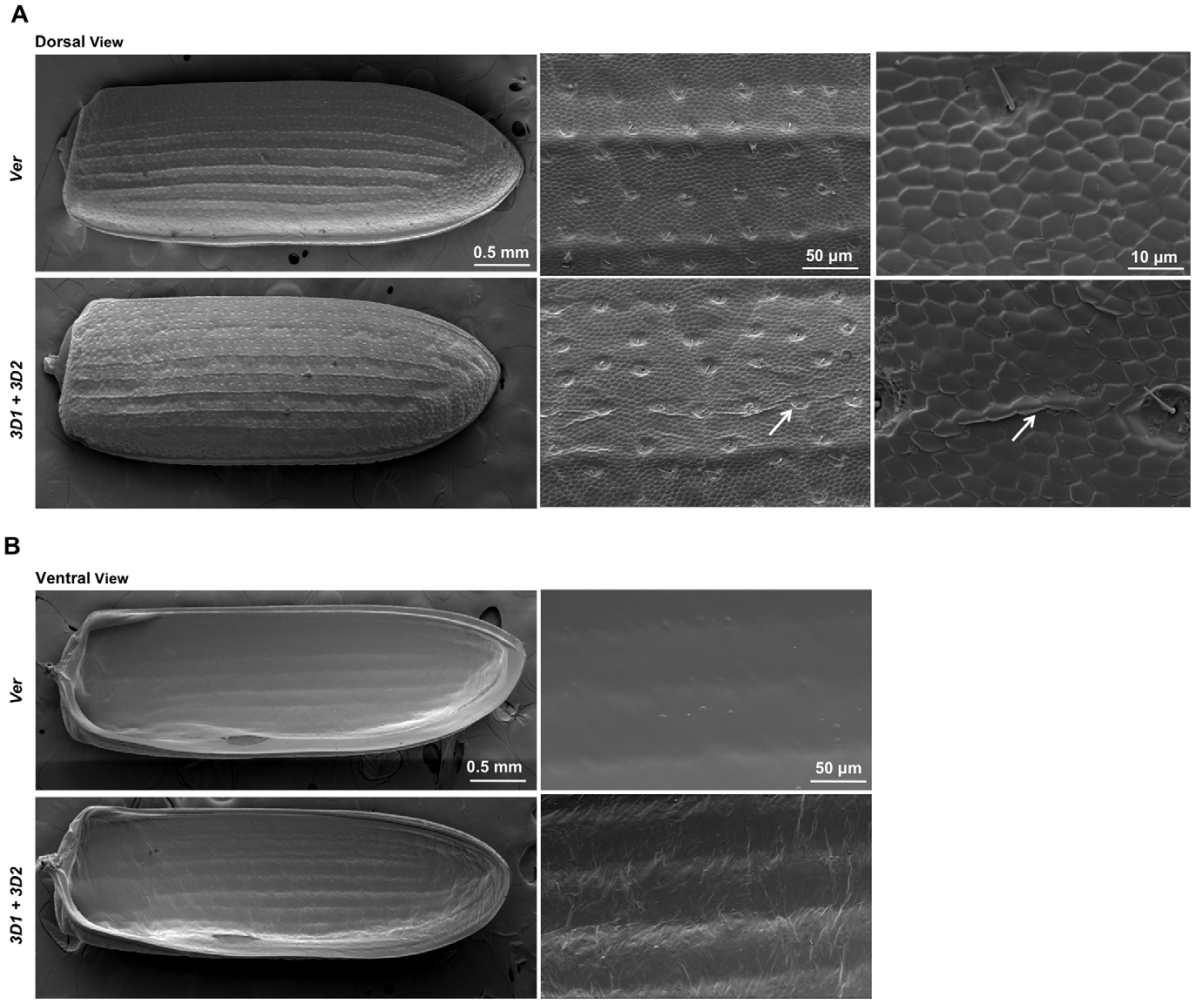
Scanning electron micrographs of pharate adult elytra from animals co-injected with dsRNA *CPAP3-D1* and dsRNA *CPAP3-D2*. dsRNAs for *TcCPAP3-D1* and *TcCPAP3-D2* were co-injected (200 ng per insect; n = 40) into penultimate instar larvae. dsRNA for *TcVer* was injected as control. Elytra were collected on pupal day 6 and analyzed by SEM. (A) Dorsal view of elytra on the top two left panels are shown at a magnification of 40 X. When compared to the dsRNA *TcVer*-injected control, co-injection of dsRNAs for *TcCPAP3-D1* and *TcCPAP3-D2* results in elytra with creases on the outer surface, which becomes more evident at higher magnification (middle panels, magnification 300 X; and right panels, magnification 2000 X). The white arrow shows the visible folds on the elytron, as it was not expanded properly. (B) Ventral view at magnification 40 X shows the rough surface of the elytron of insects co-injected with dsRNA for *TcCPAP3-D1* and *TcCPAP3-D2* (bottom left panel*)*. At higher magnification (middle panel: 300 X), the surface contains numerous unorganized fibers compared to the compact and smooth ventral surface of elytron of dsRNA *TcVer*-injected control. The chemical identity of the fibrillar structure is unknown, but it is presumed that it is chitinous.

The structural defects of the elytra were more severe when the insects were co-injected with dsRNAs for both *TcCPAP3-D1* and *TcCPAP3-D2* compared to animals injected with dsRNA for either gene alone (see Fig. 4A, 4D, 4E). Therefore, we analyzed these insects to further probe the structural details of these defects. Elytra were isolated from 10-day-old adults following co-injection with dsRNAs for *TcCPAP3-D1* and *TcCPAP3-D2* at the pharate pupal stage. These elytra were analyzed by scanning electron microscopy (SEM) and compared with elytra from animals injected with dsRNA for *TcVer*. Scanning electron microscopic analysis showed that the elytra from insects treated with both dsRNAs were not properly expanded and had creases and folds on their dorsal surfaces. The ventral surfaces of elytra from the treated animals were also rough and displayed unorganized fibrous structures (Fig. 5). The chemical composition of these fibers is unknown, but it is presumed that they are chitinous.

### CPAP Genes and Embryonic and Early Larval Development

To study the roles of *CPAP* genes in post-eclosion adult survival and reproduction, as well as on embryonic and early larval development, one-month-old adult females (n = 20 per group) were injected with 200 ng of dsRNA for each *CPAP1* or *CPAP3* gene. Adult mortality, fertility (as measured by number of eggs laid) and percent of eggs that hatched into larvae were monitored.

There was no observable effect on adult morphology or survival when dsRNA for *TcVer* was injected into adult females as a control. There was no adult mortality following adult injections of dsRNAs for any of the *CPAP* genes, with the sole exception of *TcCPAP3-A1*. Within two weeks of injection of dsRNA for *TcCPAP3-A1*, all adult females had died, exhibiting severely depleted fat bodies and abdominal tips with adhering fecal pellets (Fig. 4A, 4B).

**Figure 6:**
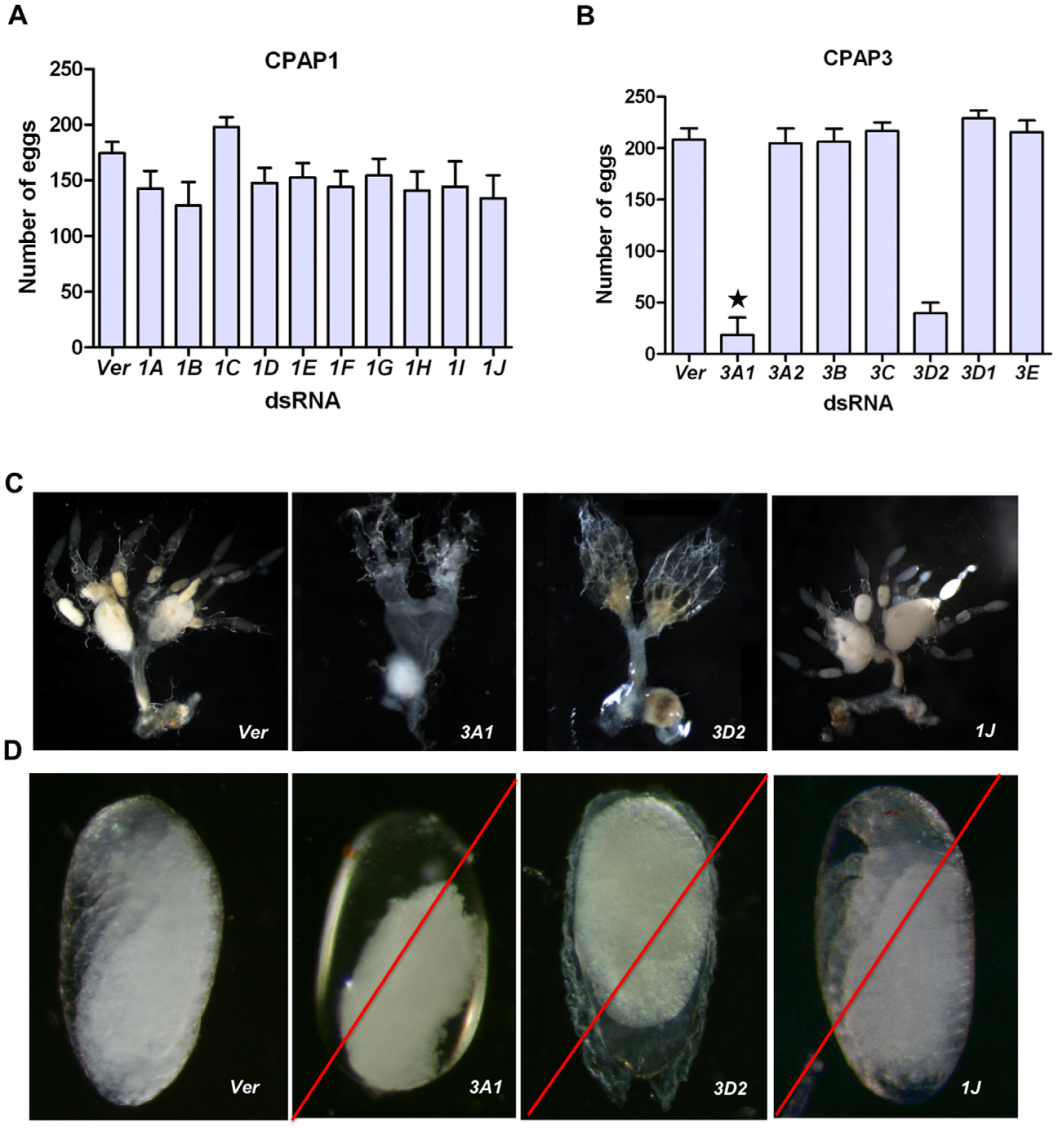
Observed RNAi phenotypes during embryonic development. (A) Comparison of the numbers of eggs laid by adult females (n = 20) following dsRNA injection for each of the 10 *CPAP1* genes and then mated with 20 males. dsRNA *TcVer* was injected as a positive control. When compared to control insects, dsRNA *CPAP1*-injected animals did not exhibit significant reduction in the number of eggs. The X-axis shows the gene target for dsRNA-treatment. Y-axis: the number of eggs laid was plotted as the mean egg number per batch +/−SD during a 3-day period. (B) Comparison of the numbers of eggs laid by a pool of adult females (n = 20) following dsRNA injection for each of the seven *CPAP3* genes. When compared to dsRNA *TcVer*-treated control insects, dsRNA *TcCPAP3-D2*-injected animals exhibited a significant reduction in the number of eggs. X-axis shows the targeted gene. Y-axis is the number of eggs laid plotted as the mean egg number per batch with SD. The asterisks indicate insufficient egg numbers due to adult lethality. (C) Dissected ovaries of dsRNA *TcCPAP3-A1*-injected females lacked proper differentiation into ovarioles and did not contain eggs. In animals treated with dsRNA for *TcCPAP3-D2,* the ovaries had ovarioles but did not contain properly developed eggs. After *TcCPAP1-J* dsRNA-treatment, the adult females had normal ovaries with eggs, similar to dsRNA *TcVer-*injected animals. (D) dsRNA for *TcVer*-injected 2-weeks-old adult female controls laid normal number of eggs and the embryos had normal body segmentation and visible appendages just before hatching. dsRNA for *TcCPAP3-A1*: In rare cases where eggs could be collected, the body segmentation was not visible presumably due to failure of embryonic development. dsRNA *TcCPAP3-D2*-treated embryos also lacked body segment differentiation and the chorion was fragile and was detached upon Clorox treatment. dsRNA for *TcCPAP1-J* treatment resulted in embryos with visible appendages and the red eye spot, but the head was not bent down like in the *TcVer* control. Instead, they held up their heads. All of these embryos showed embryonic arrest and never hatched. A red slash line indicates lethal phenotypes.

The fertility of females after injection of dsRNAs for each of the *CPAP* family genes was followed by mating a pool of twenty dsRNA-treated females with an equal number of untreated adult males, followed by collection of batches of eggs laid during several consecutive 3-day intervals. There were no significant differences in the number of eggs laid following adult RNAi for any of the *CPAP* genes with the sole exception of *TcCPAP3-D2*, which led to a drastic reduction in the number of eggs laid (Fig. 6A, 6B). We subsequently observed that the ovaries of *TcCPAP3-A1* and *TcCPAP3-D2* dsRNA-treated females were abnormal in comparison to dsRNA *TcVer* controls (Fig. 6C). The ovarioles were underdeveloped and deficient in eggs. However, the fat bodies of *TcCPAP3-D2* dsRNA-treated females were normal in appearance. The few eggs that were laid failed to hatch, and the chorion layer was fragile, easily becoming detached upon mild treatment with a 1∶1 diluted solution of 5% Clorox. The eggs contained yolk, but there was no evidence of the presence of an embryo (Fig. 6D).

Females injected with dsRNA for *TcCPAP1-J* had normal ovaries and oviposited normally, but the eggs failed to hatch. Upon dechorionation, fully developed larvae, normal in appearance and with red eye-spots, were visible under the microscope (Fig. 6D). However, germ band retraction appeared to be incomplete, because the dsRNA *TcCPAP1-J-*injected embryos placed their heads in an erect position, rather than tilted forward as observed in dsRNA *TcVer-*injected controls.

### No Phenotypes Observed after Knockdown of Other *CPAP* Family Genes

Injection of dsRNAs corresponding to *TcCPAP3-A2*, *TcCPAP3-E, TcCPAP1-A*, *TcCPAP1-B*, *TcCPAP1-D, TcCPAP1-E, TcCPAP1-F, TcCPAP1-G* or *TcCPAP1-I* at different stages of development (penultimate instar larvae, pharate pupae and pupae day-0) did not result in any observable morphological abnormalities (data not shown). Injection of dsRNAs for any of the above genes into young adult females also had no observable effect on their survival, fecundity or egg hatch.

### Chitin Analysis

#### Biochemical analysis

**Figure 7:**
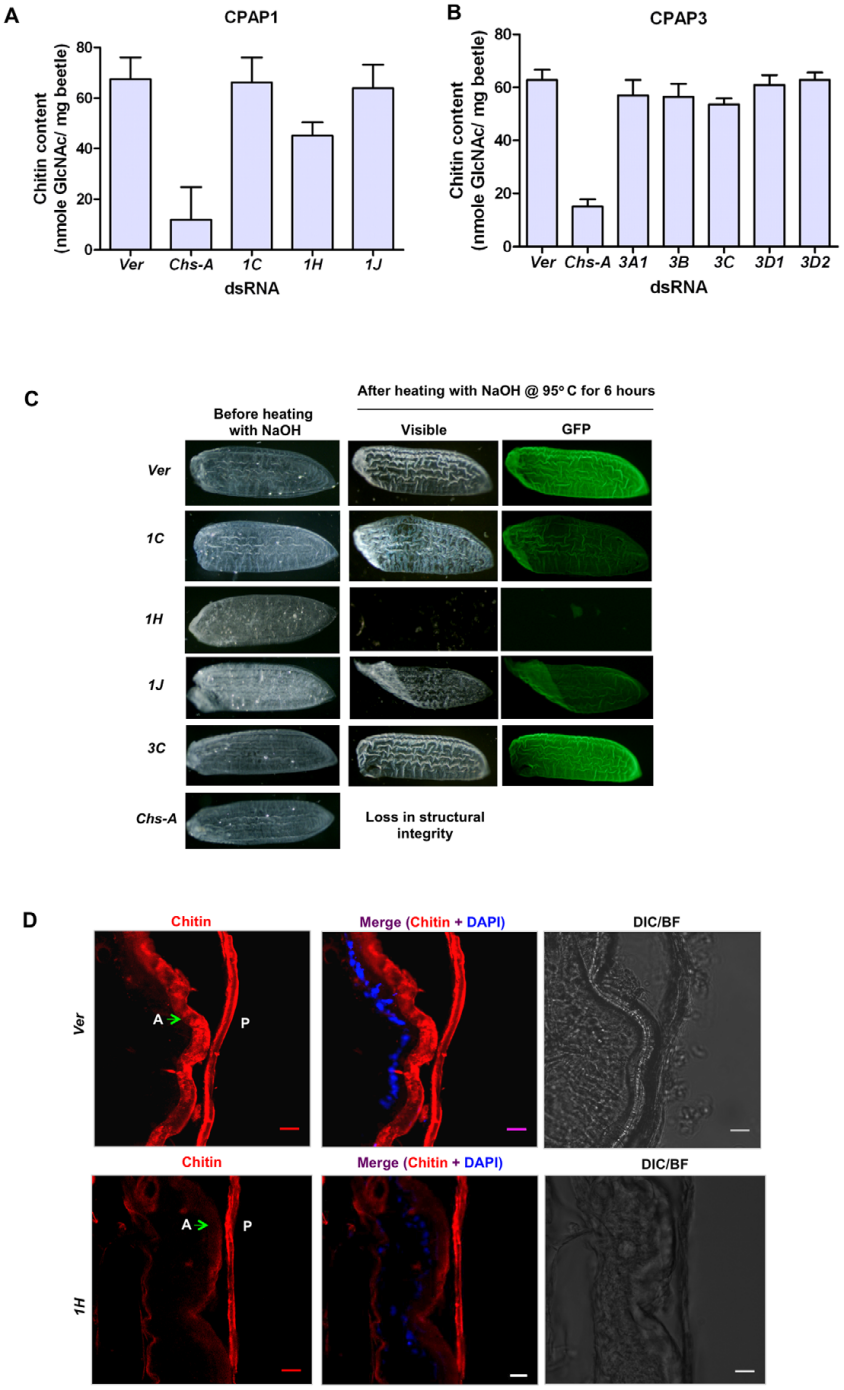
Analysis of elytra for chitin levels following RNAi for *CPAP* genes. (A and B) Biochemical analysis of chitin content of 5 day-old pupae was carried out using a modified Morgan–Elson method (n = 5) [11]. Values of mean and standard errors are shown. dsRNA for *TcVer* was injected as a control that has a normal chitin content and dsRNA for *TcChs-A* served as a control that was depleted of chitin. When compared to control insects, the dsRNA *TcCPAP1-H*-treated pupae had a slightly reduced chitin content. GlcNAc = N-acetylglucosamine. (C) FITC-CBD staining of elytral cuticle after digestion with NaOH to remove cuticular proteins. Pharate pupae were treated with dsRNAs for *TcCPAP1-C*, *TcCPAP1-H*, *TcCPAP1-J* or *TcChs-A*. The elytra were dissected out from 5-day-old pupae. The elytra from insects injected with dsRNA for *TcCPAP1-H* or *TcChs-A* were fragile and became disintegrated by the alkali treatment (10 M NaOH at 95°C for 6 hours). Elytra from dsRNA *TcCPAP1-C-* and *TcCPAP1-J*-treated insects were also fragile and lost their original shape after NaOH treatment, but they remained intact and showed less staining after treatment with FITC-conjugated chitin-binding protein (FITC-CBD) when compared with elytra from dsRNA *TcVer*-treated control insects. Elytra from dsRNA *TcCPAP3-C* treated animals did not show any effect, compared to *Ver* controls even though these insects were arrested at the adult molt. (D) Confocal microscopic analysis of pharate adult cuticle from dsRNA *TcCPAP1-H* and dsRNA *TcVer*–treated (control) insects that were stained with a rhodamine-conjugated chitin-binding probe (red). An apparent decrease in chitin staining of elytral cuticle (green arrows) following RNAi for *TcCPAP1-H* compared to RNAi for *TcVer* (control) was observed. Scale bar = 10 µm. P, pupal cuticle; A, adult elytral cuticle. Plan Apochromat objective (40 X/1.4 oil).

To determine whether the phenotypes observed after RNAi for any of the *CPAP* family genes were due to alterations in chitin content either by promoting or inhibiting chitin synthesis or by protecting chitin from degradation by chitinases, we compared chitin levels of whole animals after injection of dsRNA for the genes of the *CPAP* families that produced observable morphological defects. Chitin content of dsRNA-treated pupae collected 5 days after pupation was determined using a modified Morgan-Elson method [12], [15], [16]. When compared to the dsRNA *TcVer-*treated negative control, there was some evidence for a decrease in chitin content of whole animals following RNAi for *TcCPAP1-H*, but this reduction was not as drastic as in the dsRNA *TcChsA*-treated insects, which served as positive controls for chitin depletion (Fig. 7A, 7B). However, there was no significant difference in the chitin content of whole insects collected on pupal day 5 (the stage when molt arrest occurs) treated with dsRNA for the other *CPAP1* genes, including *TcCPAP1-C* and *TcCPAP1-J,* which also yielded lethal phenotypes. Biochemical analysis of the chitin content of animals after RNAi for the *CPAP3* genes also did not show any reduction in chitin content, unlike the case for animals treated with dsRNA for *TcChs-A* (Fig. 7A, B).

#### FITC-CBD staining

To further determine whether the reduction in chitin was confined to specific parts of the insect that are most actively synthesizing chitin, pharate adult elytra were excised on pupal day-5 and stained with a fluorescein isothiocyanate (FITC)-conjugated chitin-binding protein from *Bacillus circulans* WL-12. Before staining, the elytra were heated in 10 M NaOH at 95°C to dissolve most of the proteins, leaving a matrix consisting mostly of chitin [12]. Elytra of dsRNA *TcVer*-injected insects were used as a negative control and those from dsRNA *TcChs-A-*treated animals as a positive control for depletion of chitin. Elytra of ds*TcCPAP1-H-*treated animals were totally disintegrated after NaOH treatment, losing structural integrity, as were elytra from dsRNA *TcChs-A-*treated insects. Elytra of dsRNA *TcCPAP1-C* or dsRNA *TcCPAP1-J*-treated insects did not show any visible reduction in chitin staining. However, elytral morphology was altered after such treatment (Fig. 7C).

#### Confocal microscopy

To localize the sites of chitin depletion, confocal microscopy was performed on cryosections of dsRNA *TcVer* and dsRNA *TcCPAP1-H-*treated insects at the pharate adult stage after staining with the rhodamine-conjugated chitin-binding probe (chitin stains red). In comparison to the dsRNA *TcVer* control, we observed in *dsRNA TcCPAP1-H-*treated insects a reduction in chitin staining in the new elytral cuticle. The old pupal cuticle, however, did not exhibit a significant difference in chitin staining (Fig. 7D).

### Conclusions

### CPAP Proteins are Involved in Cuticle Organization

The main focus of this investigation has been the elucidation of the functions of members of the CPAP1 and CPAP3 families of chitin-binding proteins in *T. castaneum*. Even though the *D. melanogaster* “obstructor” family of genes (orthologs of the CPAP3 family) and their tissue-specificity of expression have been described previously [3], there have been no detailed studies on their biological functions until recently. This situation was partially remedied by Petkau et al., [7] who carried out a detailed study of one member of the *Drosophila obstructor* family of genes, namely *obstructor-A* (presumptive ortholog of *TcCPAP3-A1*; A detailed comparison of their results with those reported here is presented elsewhere in this section). Jasrapuria et al., [6] were the first to describe the *CPAP1* family of genes in insects, but there have been no reports on their physiological functions. The results presented here are from a comprehensive analysis of the functions of all members of both families of CPAP proteins, which contain either one or three peritrophin-A domains and presumably interact with chitin *in vivo*.

Several lines of evidence suggest a role for the CPAP1 and CPAP3 families of proteins in the assembly of epidermal cuticle and maintenance of its structural integrity. First, all of the CPAP proteins are predicted to have a cleavable leader peptide and lack membrane-spanning helices, and thus are expected to be secreted proteins, consistent with a role in shaping the structure of the extracellular chitin-protein matrix. Second, CPAP1 and CPAP3 proteins have one and three peritrophin-A domains, respectively, which have been implicated in chitin binding. Third, *CPAP* family genes are expressed only in epidermal tissues that synthesize cuticular chitin, and notably not in gut tissue that produces chitin required for PM assembly [6]. Finally, the *CPAP3* genes are highly expressed at developmental stages when the rate of cuticle deposition and/or tissue remodeling is elevated. This timing includes embryonic, larval and pupal stages as well as the young adult stage (Fig. 1). In addition, their expression levels generally decline in mature adult stages when new cuticle synthesis is essentially completed while PM synthesis continues unabated.

### CPAP3 Genes have Diverse Roles in Molting, Locomotion and Egg Hatching

In this work we have used RNAi, which is systemic and splice-variant-specific in *T. castaneum*, to investigate the roles of individual *CPAP1* and *CPAP3* genes in this species at multiple developmental stages and in different cuticle-forming tissues. These RNAi experiments have revealed that many of the *CPAP3* genes are indeed indispensable for insect survival or maintenance of normal morphology and cuticle integrity. Among the seven genes in the *T. castaneum CPAP3* family, RNAi for five genes (including the two alternative exons of *TcCPAP3-C*), individually or in some combinations, yielded a large spectrum of phenotypes including molt arrest and lethality, elytral abnormalities, defects in articulation of joints, abnormal gait, reduction in fecundity, fat body depletion, underdeveloped ovaries, reduced egg hatching and adult mortality.

Table 2 summarizes the results of RNAi for *TcCPAP3* genes, including many that yielded observable phenotypes. Of the seven genes, RNAi for only two genes resulted in mortality either at the pharate adult stage (*TcCPAP3-C*) or at the young adult stage (*TcCPAP3-A1*). RNAi for three other *CPAP3* family genes (*TcCPAP3-B, TcCPAP3-D1* and *TcCPAP3-D2*) led to cuticle abnormalities, the most obvious ones being wrinkled elytra and a dimpled pronotum. The finding that orthologs of all of these *CPAP3* genes are found in insect genomes of different orders [6], [7] further strengthens our conclusion from RNAi studies that many members of the CPAP3 family of proteins serve distinctly different but essential functions. Expression data for the *Obstructor* (*CPAP3*) family of genes of *D. melanogaster* also indicate that many of these genes are expressed in larval tracheae and carcass (e.g. http://flybase.org/reports/FBgn0031097.html/FBgn0027600.html/FBgn0026077.html). The ortholog of *TcCPAP3-A1* in *D. melanogaster* has been shown to be essential for larval growth, molting, tracheal tube expansion and wound healing [7]. The protein product of this gene is indeed detectable at high levels in larval and pupal stages, and at lower levels in adults.

**Table 2:** Summary of RNAi for *CPAP1* and *CPAP3* genes in *T. castaneum*.

CPAP1			
Gene	Protein Accession #	RNAi phenotype	Affected tissue
*TcCPAP1-A*	ACY95466	None detected	–
*TcCPAP1-B*	ACY95467	None detected	–
*TcCPAP1-C*	ACY95468	PA-A molting (lethal)	Epidermal cuticle
*TcCPAP1-D*	ACY95469	None detected	–
*TcCPAP1-E*	ACY95470	None detected	–
*TcCPAP1-F*	ACY95471	None detected	–
*TcCPAP1-G*	ACY95472	None detected	–
*TcCPAP1-H*	ACY95473	PA-A molting (lethal)	Epidermal cuticle, Elytra
*TcCPAP1-I*	ACZ04319	None detected	–
*TcCPAP1-J*	ACY95474	PA-A molting (lethal)	Epidermal cuticle, Elytra, Embryo development
**CPAP3**			
**Gene**	**Protein Accession #**	**RNAi phenotype**	**Affected tissue**
*TcCPAP3-A1*	ACY95475	Adult lethality	Ovaries, Hindgut, Fatbody
*TcCPAP3-A2*	ACY95476	None detected	–
*TcCPAP3-B*	ABL73928	Walking defect	Stiff leg joints
*TcCPAP3-C*	ABL73929	PA-A molting (lethal)	Epidermal cuticle
*TcCPAP3-D1*	ACY95477	Rough adult elytra	Elytra
*TcCPAP3-D2*	ABL73931	Rough adult elytra	Elytra, Embryo development
*TcCPAP3-E*	ACY95478	None detected	–

Insects (n = 40) were injected with double-stranded RNAs (200 ng per insect) at different developmental stages as mentioned in the Materials and Methods section, the resulting phenotype and the affected tissue, if any has been shown. PP, pharate pupae; A, adult.

The finding that down-regulation of the expression of *CPAP3-C* leads to molting arrest and death at the pharate adult but not during earlier stages of development of *T. castaneum* suggests that this protein may be indispensable only during pupal-adult metamorphosis. The absence of any detectable phenotype following RNAi for *CPAP3-C* at earlier developmental stages, when this gene is indeed expressed, suggests that other CPAP3 proteins may compensate for the absence of this protein in the formation of the larval and pupal cuticles. Parental RNAi experiments indicated that down-regulation of this gene also did not appear to affect embryonic development, egg hatch or larval viability, indicating that this gene is not essential during embryonic development. *TcCPAP3-C* gene is the ortholog of the “*gasp*” or “*obstructor C”* gene previously reported in *D. melanogaster* [3], [5]. *Obstructor-C*, which is expressed in larval trachea, carcass and hindgut is among the proteins that are secreted into the tracheal lumen and reabsorbed by lumenal cells during tubule clearance and air-filling [17]. However, no mutant phenotypes have been reported for this gene in *D. melanogaster* or any other insect. The finding that among the seven members of the *T. castaneum CPAP3* gene family, RNAi for this gene alone leads to developmental arrest and death prior to the adult stage indicates an essential role for this gene in development.

In contrast to the results of RNAi for *TcCPAP3C*, which led to molting arrest at the pharate adult stage, RNAi for *TcCPAP3-A1* at larval or prepupal stages did not affect molting, larval growth or survival, cuticle morphology or adult emergence, but it did lead to mortality one week after adult eclosion. RNAi of adult females also resulted in death within two weeks after dsRNA administration. The observation that these animals had depleted fat body tissue and were unable to defecate normally suggested that a cuticle lining the digestive system such as that of the hindgut or foregut might have been affected in these animals. Knocking down transcripts of both *TcCPAP3-A1* and *TcCPAP3-A2* simultaneously did not further enhance mortality or result in molting defects. RNAi for *TcCPAP3-A2* alone did not result in any abnormal phenotype, indicating that *TcCPAP3-A2* may not be an essential gene. However, its higher expression in the elytron relative to the wing tissue during the pupal stages [6] suggests that it may have a role in shaping the elytral cuticle at the ultra-structural level. This remains a subject for future study. It is interesting to point out that null mutants of *Drosophila obstructor-A*, the ortholog of *TcCPAP3-A*, exhibited greatly retarded larval growth, multiple cuticular abnormalities, convoluted tracheal tubules and defective chitin removal from tracheal lumen. Such insects died in early larval stages in spite of consuming food [7]. In *T. castaneum*, we did not find any obvious cuticular abnormalities by carrying out RNAi of *TcCPAP3-A1* at the fourth and fifth larval instars and prepupal stages. The larvae (or prepupae) developed into mature adults, but within a week after adult eclosion, they had depleted fat bodies, and died showing obvious defecation problems precluding studies of effects on oogenesis, oviposition, embryonic and/or early larval development. The reason for the difference in the effects of loss of function of this gene in these two insect species is unclear. One possible explanation that the presence of the paralogous gene, *TcCPAP3-A2,* in *Tribolium* and other insect species [6], [7] (unlike *Drosophila,* which has only a single *TcCPAP3-A*-like gene), has redundant and compensating functions seems unlikely as down-regulation of transcripts of both of these genes does not result in lethality at earlier developmental stages. We have not performed RNAi using combinations dsRNAs for multiple *CPAP3* genes, which may have redundant functions. However, two transcripts of *Obstructor-A* that differ only in the length of the 3′-UTR region have been described in *D. melanogaster* [7]. Whether or not these two alternatively polyadenylated transcripts of *Obstructor A* serve distinct functions in different tissues or developmental stages of *D. melanogaster* has not been determined. The protein products of this gene are present at high levels in late embryos, and pupae and persist in the adult stage in *D. melanogaster* suggesting that this gene is functional through all stages of insect life [7]. Similar experiments have not been conducted with *T. castaneum* due to the non-availability of specific antibodies to address the differences in the requirements for these gene products during development in *Tribolium* and *Drosophila*.

While *TcCPAP3-D1* and *TcCPAP3–D2* are dispensable for molting and adult survival of *T. castaneum*, insects subjected to RNAi for either of these genes have clearly visible deformities in the elytron or pronotum, suggesting that some aspect of cuticular structure has been altered. RNAi using a mixture of dsRNAs for *TcCPAP3-D1* and *TcCPAP3–D2* resulted in synergistic effects (more pronouncedly wrinkled elytra), indicating these may be recently duplicated genes with similar functions. Nonetheless, after RNAi for *TcCPAP3-D1* and *TcCPAP3-D2*, we could observe defects in the elytral cuticle with the severity increasing when transcript levels of both of these genes were down-regulated, indicating that these two CPAP proteins may be important for maintaining the structural integrity of the elytra. SEM experiments provided strong evidence that the organization of the elytral cuticle is affected following RNAi for these two genes because we observed creases and unorganized fibers in the elytra compared to dsRNA *Ver*-treated control insects.

Similarly, down-regulation of the levels of *TcCPAP3-B* transcripts did not affect molting or even adult survival for at least a month, but these animals had split elytra and dimpled pronota. Most remarkably, they exhibited defective locomotor ability, possibly indicating a failure of normal articulation of the femoral-tibial and tibial-tarsal leg joints. The wobbly gait observed after RNAi for *TcCPAP3-B* suggests that this protein may influence the flexibility of these joints. Thus, a subset of *TcCPAP3* family genes is nonessential for survival but is critical for normal functioning of specific types of cuticles.

### Only Three Members of the CPAP1 Family are Essential for Development and Cuticle Integrity

The functions of the *CPAP1* family of genes are not obvious because there is no information in the databases about the nature of the domains of these proteins except for the peritrophin-A domain. Three genes belonging to the *CPAP1* family, *TcCPAP1-C, TcCPAP1-H* and *TcCPAP1-J*, appear to be essential, based on lethality at the pharate adult stage following RNAi. Confocal microscopy confirmed that RNAi for at least one of these, *TcCPAP1-H*, is associated with a detectable loss of chitin in the newly formed adult elytral procuticle but not in the pupal cuticle. The causes of death at the pharate adult stage following RNAi of *TcCPAP1-C* and *TcCPAP1-J* are unclear, but there is extensive tissue remodeling occurring at this stage and some of these processes could require specific *CPAP1* genes. While the functions of the other CPAP1 family proteins could not be demonstrated by RNAi, they may have important functions because orthologs for these genes are present in several orders of insects including *D. melanogaster* (Table 1).

### Matrices with Different Compositions of CPAP Proteins Display Divergent Physicochemical Properties

It is likely that the nature of the chitin-binding proteins as well as the cross-linking of these proteins to chitin and other proteins influence the properties of the cuticle and PM, which range from membranous (PM) to flexible (e.g. wing) to rigid (e.g. larval head capsule and elytron). In the cuticle, these variations are likely to result in part from different CPAP proteins being incorporated into different cuticles. It has been proposed that hard cuticle is more generally associated with proteins that contain the RR2 motif, whereas soft cuticle is associated with proteins that contain the RR1 motif [1], [18], [19]. Knock-down of transcripts for two cuticular proteins that are abundant in elytra of *T. castaneum* belonging to the RR2 family has been shown to result in less rigid elytra with altered mechanical properties and also in mortality of insects a week after adult eclosion [20].

The present study has established that specific members of either the CPAP1 or CPAP3 family influence the physicochemical properties of the cuticle. Elytra isolated from some of the RNAi experiments described in this work (e.g. *TcCPAP1-H*) are fragile and deficient in chitin as reported for elytra from animals depleted of chitin by treatment with dsRNA for *TcChs-A* [12] or dsRNA for Knickkopf, (Knk*),* a protein essential for chitin organization in the cuticle [21]. Elytra from animals with reduced expression of *TcCPAP1-J* or *TcCPAP1-C* were also fragile and had lost most of their structural integrity, whereas down-regulation of transcripts for other genes such as *TcCPAP3-C* did not result in such a phenotype, even though all three of these phenotypes had nearly the same amount of chitin as control animals. Thus, many and perhaps all CPAP proteins may play unique roles in constructing specific types of cuticle at specific anatomical locations such as joints and head skeleton, which in turn may influence the unique mechanical properties of each type of cuticle.

### Some CPAP Proteins Affect Cuticle Integrity by Affecting Content and/or Organization of Cuticular Chitin

The most common defect seen in the RNAi experiments reported here was developmental arrest at the time of adult molting when there is extensive tissue remodeling and cuticle deposition. Rough and shortened elytra as well as a defective gait might be secondary consequences of the cuticular defects. We suspect that even though the chitin content of many cuticles was unaffected, one or more of other structural parameters including the antiparallel organization of the chitin microfibers, the organization of these fibers into macrofibers and laminae, and the stacking of these laminae into the helicoidal or pseudoorthogonal structures as described for other arthropods [22] may have been affected in our RNAi experiments. Loss of laminar architecture by depletion of the cuticle-organizing protein Knk, has been demonstrated in *D. melanogaster* and *T. castaneum* [21], [23]. This protein also protects chitin from degradation by chitinases [21], [24]. Whether CPAP proteins play similar roles in protecting or maintaining exoskeletal chitin in specific cuticular structures remains to be determined.

The requirement for expression of some *CPAP* genes for survival or fecundity during the adult stage when most of the body wall and appendage-associated cuticle is fully assembled indicates that there is turnover of some cuticular chitin, perhaps involving the digestive tract or the reproductive tissues. Two genes, *TcCPAP1-J* and *TcCPAP3-D2,* which are essential for survival at the pharate adult stage, affect fecundity or egg hatch. The defective egg maturation that was seen in ovarioles following RNAi for *TcCPAP3-D2* may be due to some structural abnormality of the ovaries and/or eggshell. Indeed eggs, ovaries and egg shells of *Aedes aegypti* have been shown to contain chitin-like material [24]. Following parental RNAi for *TcCPAP1-J*, the females oviposit normally and the embryos are fully developed but fail to hatch. The larval head is tilted upward rather than tucked down ventrally as in normal or dsRNA *Ver*-treated controls, possibly indicating incomplete retraction of the germ band. This phenotype is different from that reported following RNAi for *TcChs-A,* in which the embryos are misshapen and have a significant reduction in chitin content [11]. The *TcCPAP3-D2* gene also appears to be involved in some early developmental events, because there is no evidence of embryonic development after parental RNAi for this gene.

In conclusion, differences in the developmental expression profiles of individual *CPAP3* and *CPAP1* genes and differences in expression in different cuticle-forming tissues (e.g. elytron versus hindwing), as well as the diverse phenotypes resulting from down-regulation of individual genes of the *CPAP* family, suggest that many of these genes have distinct, non-redundant functions. This hypothesis is further supported by our finding that down-regulation of individual transcripts or splice variants results in lethality and/or morphological defects, which, in most cases, cannot be compensated for by other members of the same family. Since all of the encoded proteins probably bind to chitin, they could interact with this matrix polymer and/or other cuticular proteins in distinctly different ways. One can speculate that specific CPAP proteins are involved at different levels of organization of chitin into nanofibrils, larger microfibrils of chitin-protein complexes, laminae and stacked laminae. The presence of three ChtBD2 domains in the CPAP3 family of proteins makes them particularly attractive candidates to be involved in non-covalent and/or covalent interactions with chitin and other proteins in nearby fibrils. The CPAP1 family proteins, which have other domains in addition to a single peritrophin-A domain, appear to be ideal candidates for either protein-protein interactions or for other uncharacterized enzymatic functions in a chitin-rich environment as has been shown for many chitinases and chitin deacetylases, some of which have one or more peritrophin-A domains [25]. In these enzymes, the chitin-binding domain probably helps to anchor the enzymes onto the insoluble chitin matrix, facilitating performance of their enzymatic functions by preventing premature dissociation from the chitin substrate. It is conceivable that uncharacterized proteases or other enzymes may utilize their built-in chitin-binding domains to immobilize themselves in the chitinous cuticle.

## Supporting Information

Table S1 **Summary of properties of dsRNAs used for RNAi studies.** The table provides the nucleotide position, the length in base pairs of the amplicon and the number of dsRNAs targeted for each of the *TcCPAP1* and *TcCPAP3* genes.
(XLSX)

Video S1 **dsRNA** ***TcCPAP3-B*** **-injected insects show uncoordinated gait, stiff-joints and split elytra.** The pronota were also dimpled.
(MOV)
